# *Porphyromonas gingivalis*, a Long-Range Pathogen: Systemic Impact and Therapeutic Implications

**DOI:** 10.3390/microorganisms8060869

**Published:** 2020-06-09

**Authors:** Hannah Mulhall, Olivier Huck, Salomon Amar

**Affiliations:** 1 Department of Microbiology and Immunology, New York Medical College, Valhalla, NY 10595, USA; Hannah_Mulhall@student.nymc.edu; 2 Department of Pharmacology, New York Medical College, Valhalla, NY 10595, USA; Huck.Olivier@gmail.com; 3 Faculté de Chirurgie-Dentaire, Université de Strasbourg, 8 rue Sainte-Elisabeth, 67000 Strasbourg, France; 4 INSERM (French National Institute of Health and Medical Research), UMR 1260, Regenerative Nanomedicine, Fédération de Médecine Translationnelle de Strasbourg (FMTS), 67000 Strasbourg, France

**Keywords:** periodontitis, inflammation, systemic interactions, infection, dysbiosis, microbiome

## Abstract

Periodontitis is an inflammatory disease associated with a dysbiosis of the oral flora characterized by a chronic sustained inflammation leading to destruction of tooth-supporting tissues. Over the last decade, an association between periodontitis and systemic disorders such as cardiovascular diseases, rheumatoid arthritis and obesity has been demonstrated. The role of periodontal pathogens, notably *Porphyromonas gingivalis* (*P. gingivalis*), in the onset or exacerbation of systemic diseases has been proposed. *P. gingivalis* expresses several virulence factors that promote its survival, spreading, and sustaining systemic inflammation. Recently, the impact of periodontitis on gut dysbiosis has also been suggested as a potential mechanism underlying the systemic influence of periodontitis. New therapeutic strategies for periodontitis and other dysbiotic conditions, including the use of beneficial microbes to restore healthy microbial flora, may pave the way to improved therapeutic outcomes and more thorough patient management.

## 1. Introduction

Periodontitis is an oral inflammatory disease of infectious origin affecting *periodontium*, characterized by gingival swelling and bleeding, periodontal pocket formation and clinical attachment loss, destruction of tooth-supporting tissue and ultimately to tooth loss [1,2]. Periodontitis is among the most common diseases worldwide, with its severe form affecting approximately 11% of the global population [3]. This disease represents a major public health issue as periodontitis has been correlated with decreased oral health and related quality of life [4]. Furthermore, an association with several systemic diseases such as cardiovascular diseases, diabetes and rheumatoid arthritis was established [5]. The oral microbiome is a highly complex ecosystem with thousands of microbial phylotypes detected [6], and its understanding presents a challenge. In the context of periodontal inflammation, the contribution of microbiome dysbiosis is under investigation due to the molecular mechanisms underlying this microbiome state being poorly understood.

Microbiome dysbiosis is characterized by an imbalance between microbial species and their environment. The imbalance seems to be driven by the subversion of host immunity due to keystone pathogens even when they are present in low abundance [7,8]. Typically, an inflammatory host response would lead to the clearance of pathogenic bacteria. However, in the case of periodontal disease, it promotes the growth of keystone pathogens due to gingival inflammatory exudate being a rich source of nutrients [9]. Keystone pathogens, such as *Porphyromonas gingivalis* (*P. gingivalis*), facilitate the subversion of the host immune response through polymicrobial synergy allowing for the growth of both themselves and other dysbiotic species [10,11,12]. Polymicrobial synergy within the pathogenic oral biofilm is critical for the establishment and spread of periodontal disease. It has been demonstrated in vitro that there is a cooperative biofilm formation involving *Streptococcus gordonii*, *Fusobacterium nucleatum* (*F. nucleatum*) and *P. gingivalis* [12]. When cocultured, these bacteria elicited extensive changes on each other’s biochemical signatures to support cohabitation within the biofilm [13]. During colonization, *F. nucleatum* triggers the activation of nicotinamide adenine dinucleotide phosphate (NADPH) oxidase in the host cells which provides a more favorable environment for *P. gingivalis* attachment [14]. Furthermore, this leads to the upregulation of virulence factor, mfa1 fimbriae, in both *F. nucleatum* and *P. gingivalis* preventing dendritic cell maturation allowing the disruption of the innate immune response [12].

*P. gingivalis* deploys an extensive arsenal of virulence factors such as lipopolysaccharide, proteases, fimbriae and a CRISPR-Cas system [8,15,16]; enabling it to modulate the host immune response to promote its survival through cellular colonization and spreading [17,18]. During the initial phase of infection, this bacterium manipulates the immune system through inhibition of cytokines and chemokines secretion [19,20]. Neutrophil homing to the gingival tissues is critical for maintaining homeostasis between the host and the microbiome. *P. gingivalis* creates a chemokine paralysis by degrading IL-8 through its secreted gingipain proteases and prevents IL-8 transcription through SerB, a haloacid dehalogenase (HAD) family serine phosphatase [21,22]. Furthermore, the ability of *P. gingivalis* to persist in the periodontal tissue after chemokine paralysis may depend to its ability to hijack the complement system, preventing its clearance from the oral cavity [11,23,24]. In addition to paralyzing the immune response, *P. gingivalis* also targets other periodontal cell types such as gingival epithelial cells, fibroblasts, periodontal ligament cells, and osseous cells, leading to the establishment of an inflammatory environment [17,18,25,26,27]. This hijacking of the host immune response hinders immune cell recruitment, allowing *P. gingivalis* to spread and colonize the periodontal pocket.

## 2. Distant Dissemination of *P. gingivalis*

**Figure 1:**
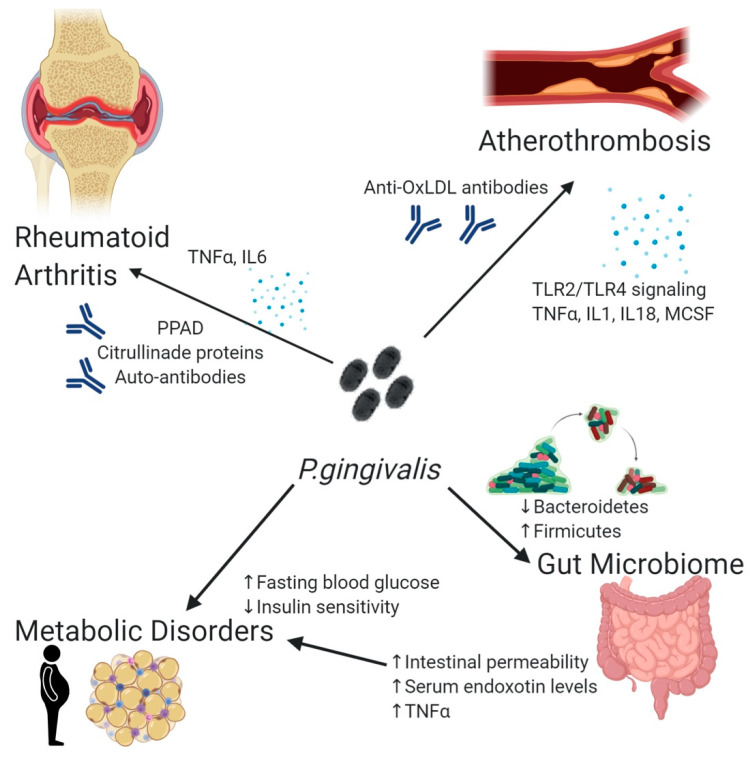
*Porphyromonas gingivalis* influences the development of multiple chronic inflammatory conditions. Through the cross-reactive antibodies (atherothrombosis, rheumatoid arthritis), increased levels of systemic inflammation (atherothrombosis, rheumatoid arthritis, gut microbiome dysbiosis, metabolic disorders), as well as overall microbiome dysbiosis. (↑ = increase ↓= decrease).

Over the last decade, there has been increased interest on the links between periodontitis and systemic diseases [5]. Among them, an association has been demonstrated with major chronic diseases such as cardiovascular diseases [28], diabetes [29], metabolic syndrome [30], rheumatoid arthritis [31] and more recently obesity [32] (Figure 1). Interestingly, periodontitis has been documented as contributing to the risk of all-cause mortality in an older European population (subjects 60–70 years of age; hazard ratio = 1.57 (1.04–2.36) after adjustment for confounding variables) [33]. Therefore, the systemic effects of oral pathogens and the role they play in chronic diseases has become a major research focus.

Among the oral bacteria that exhibit systemic effects, *P. gingivalis* has stood out. It has been detected in several diseased tissues and organs in both humans and animal models. The translocation of *P. gingivalis* to the distant tissues such as the liver or joint after oral administration [34,35] and its detection in brains of patients with Alzheimer’s disease [36] has led to an increased interest in determining its role in chronic inflammatory diseases.

## 3. Pathogenicity of *P. gingivalis*: The Case of Atherothrombosis

Chronic infection has been identified as a potential contributor to the development of atherosclerotic lesions independent of classical risk factors such as unfavorable lipid profile [37,38]. *Chlamydia pneumoniae*, *Helicobacter pylori*, *P. gingivalis* and certain viruses have been detected within atheromatous plaque [39,40,41]. However, their impact remains under investigation. Several periodontal pathogens have been detected in both atherosclerotic plaque and healthy vessels [41,42]. *P. gingivalis* is among the most commonly found organism in these studies due to its ability to persist within vascular tissue through cell-to-cell transmission [43].

The severity of periodontitis in experimental models has been shown to correlate with the magnitude of the systemic inflammation as well as atheromatous plaque formation. Oral administration of *P. gingivalis* has been reported to accelerate the development of atherosclerosis in apolipoprotein E knock out (Apoe−/−) mice [38,44,45]. In the experimental models of periodontitis, it has been demonstrated that there is an increased systemic inflammation, potentially contributing to vascular lesion development [46,47]. Signaling through TLR-2 and TLR-4 is critical for the development of periodontitis, as well as atherosclerotic plaque progression [48]. It has been demonstrated that *P. gingivalis* is able to activate these membrane receptors on the endothelial level [49,50] triggering the secretion of cytokines such as TNF-α, IL-1, IL-18 and M-CSF [51] thus, contributing to a persistent inflammation. Furthermore, in these mice, *P. gingivalis* DNA can be found in the aortic tissue along with an abundance of activated macrophages [44,45]. Interestingly, when nonsurgical periodontal therapy is performed in these models, there is a reduction in systemic inflammation as well as aortic inflammation supporting the causative role oral microbiome dysbiosis [52].

Oxidized low density lipoproteins (OxLDLs) are believed to initiate the immunological response found in atherosclerosis [53]. It has been demonstrated in patients with stable coronary artery disease, as well as acute coronary disease, that antibodies directed against key virulence factors of *P. gingivalis* can cross-react with OxLDLs, malondialdehyde-modified low-density lipoprotein (MDA-LDL) and to malondialdehyde acetaldehyde-modified low-density lipoprotein (MAA-LDL) [54]. In vitro, the synergic effects between risk factors have also been observed. Indeed, *P. gingivalis*-induced effects can be influenced by the cellular environment and other proinflammatory triggers, such as oxidized low-density lipoproteins, (Ox-LDL) [55] highlighting the fact that vascular inflammation could arise from different mechanisms and insults. The antibody cross-reaction between bacterial components and host molecules may also contribute to the inflammatory environment in the atherosclerotic plaque. In mouse models, immunization with *P. gingivalis* or its gingipain, Rgp44, seems to display athero-protective effects in the modulation of plaque size, and anti-inflammatory cytokines IL-10 and IL-5 [54].

Based on the cumulative evidence strengthening the role of bacterial infection in atherosclerosis, several clinical trials have been conducted assessing the impact of antibiotic administration on atherosclerosis-related parameters in patients with stable coronary artery disease (WIZARD, ACES, CLARICOR), acute coronary syndrome (PROVE-IT-TIMI) and peripheral artery disease (PROVIDENCE-1) [56,57,58,59,60]. However, none of these trials demonstrated long term benefit of such a therapeutic strategy. These results might be explained by the fact that 1. antibiotics never penetrate a biofilm unless mechanically disrupted; 2. antibiotics must be able to address host cell invasion by pathogens; 3. the poorly designed studies focus mainly on late stages of atherothrombosis, reducing the ability to detect a benefit of such an approach [61]. Future trials testing the effect of antibiotics on earlier stage disease might be worth consideration.

## 4. *P. gingivalis* and Rheumatoid Arthritis

Rheumatoid arthritis (RA) is a systemic autoimmune inflammatory disease that primary affects the joints, however, the condition can damage a wide variety of body systems including the skin, eyes, lungs, heart and blood vessels [62]. The notion that RA had an infectious origin is not a new one. Since the early 19th century, investigators have claimed to identify different bacteria in RA synovial fluid and tissue. Although these results were not reproducible, the evidence supported the theory that RA was a result of microbial action either within the joint or systemically [63]. The association between periodontal disease and RA has long been established [64]. Initially, it was thought that the alveolar bone destruction and gingival inflammation was part of the same RA-induced damage. It was not until recently that periodontitis was seen more as a cause than a consequence of RA. Like periodontitis, the tissue damage seen in RA is due to a host driven inflammation [65,66]. The cytokine signature of each disease is similar with an increased expression of proinflammatory cytokines, TNF-α and IL-6. In murine models of experimental arthritis, it was found that there is an increase in the serum levels of these inflammatory mediators following oral administration of periodontal pathogens, supporting their role in exacerbating the local and systemic inflammation associated with RA [67,68,69]. Additionally, auto-antibodies such as rheumatoid factor and anticollagen antibodies common to RA are often found in diseased periodontal tissues [70,71]. Specific periodontal pathogens have even been identified as risk factors for the development of RA, and *P. gingivalis* in particular has received a lot of attention. *P. gingivalis* secretes a petidylarginine deiminase (PAD)-like enzyme (PPAD), which can contribute to the generation of citrullinated proteins, a major target of autoantibodies in RA [72,73]. PPAD is secreted in a soluble form and in association with the outer membrane vesicles of *P. gingivalis* [74]. As a major virulence factor, PPAD helps to maintain the presence of *P. gingivalis* in the mouth by preventing neutrophil function as well as the production of ammonia [75,76,77]. Clinical isolates of *P. gingivalis* have been shown to be highly enriched in citrullinated proteins due to the functioning of PPAD as well as its gingipain Rgp [73,78]. In murine models of collagen-induced arthritis, inoculation with *P. gingivalis* lead to an increase in citrullinated peptides as well as an increased expression of proteins targeted for citrullination [78]. The production of bacterial and host citrullinated proteins by PPAD may further exacerbate the loss of tolerance to citrullinated autoantigens in RA. In murine models of collagen-induced arthritis PPAD-null mutants, *P. gingivalis* does not elicit the same level of inflammation or autoantibodies to collagen type II, highlighting its potential for therapeutic targeting [73]. In addition to autoreactive antibodies, patients with RA often have high levels of antibodies to *P. gingivalis* gingipain domains [79]. A majority of the antigingipain antibodies target the hemagglutinin (HA2-4) as well as the catalytic domains [80]. Interestingly, in the murine model of collagen-induced arthritis, rats that received a pretreatment vaccination of the catalytic domain of RgpA had less severe cartilage erosive changes when compared to controls. Furthermore, rats that received the catalytic domain of RgpA demonstrated a reduction in inflammatory mediators (IL-1b and TNF-α) both at the mRNA and protein level in the synovial tissues [79].

It is difficult to clearly define periodontitis and periodontal pathogens as microbial cause of RA, given 11% of the global adult population present periodontitis and RA affects only 0.5 to 1% [3,81]. However, there is overwhelming evidence to support that periodontal pathogens, specifically *P. gingivalis*, play a role in the progression of the disease by allowing for the accumulation of inflammatory cytokines and autoreactive antibodies, leading to the tissue destruction associated with RA [69,82,83,84]. Furthermore, it has been demonstrated in numerous animal models of experimental arthritis that arthritis can be treated by targeting *P. gingivalis* and its numerous virulence factors, supporting the need for further investigation into the effects of nonsurgical periodontal treatment and the progression of RA in human trials.

## 5. Impact of *P. gingivalis* on Obesity, Metabolism, and Gut Microbiota Homeostasis

The amount of data illustrating a relationship between obesity and periodontitis [85] has led to interest in the potential role played by *P. gingivalis* on both sustained inflammation in adipose tissue and gut microbiome-associated changes. It has been suggested that bacterial insult may contribute to obesity-related chronic inflammation and oxidative stress [86]. Endotoxemia characterized by high plasma level of bacterial lipopolysaccharides (LPS) has been shown to stimulate adipocyte proliferation and inflammation in adipose tissue [87]. Interestingly, infection of macrophages by *P. gingivalis* induced a switch in macrophage polarization leading to the inhibition of M1 phenotype, illustrating an alteration of immune response with effects on a cascade of pathways associated with immune cell proliferation and angiogenesis. These effects are potential causes of the delay in the response to infection observed in obese individuals [88]. However, it should be mentioned that a sole bacterium cannot cause metabolic endotoxemia and that the establishment of the low-grade inflammation is the result of a complex process that involves the whole microbiota, including butyrate-producing bacteria, mucin-degrading bacteria and LPS-containing bacteria such as *P. gingivalis* [87,89,90,91].

The gut microbiome has recently come to be viewed as an organ system in its own right [92]. It is the most diverse commensal ecosystem in the human body that continues to evolve throughout the human lifespan [93]. Microbes residing in the gut are considered key contributors to host metabolism as they interact with virtually all human cells and induce significant changes in host metabolism [89]. Metabolic disorders, such as obesity, are associated with shifts in the microbiota at the phylum level, especially in the Firmicutes/Bacteroidetes ratio [94]. Saliva is an important transporter of oral bacteria to the gut as daily saliva production is approximately 0.6 L [95]. Clinical trials have demonstrated that bacterial counts of periodontal pathogens in saliva (*Campylobacter rectus*, *F. nucleatum*, *P. gingivalis*, *Prevotella intermedia* and *Tannerella forsythia*) were increased with the severity of periodontal disease (gingivitis/periodontitis) [96]. It has been hypothesized that swallowed oral pathogens can lead to disturbances in the gut microbiota causing metabolic endotoxemia and metabolic disorders [97]. Patients with chronic periodontitis have been shown to have less diversity in their gut microbiomes [98]. Several studies have investigated the influence of *P. gingivalis* oral gavage on the gut microbiome, mostly in animal models. All studies indicate a significant modification of gut microbiome composition after *P. gingivalis* oral administration [99,100]. In C57BL/6 mice infected with *P. gingivalis* strain W83, Kato et al. showed that the proportion of Bacteroidetes was significantly reduced compared to sham-administered mice, indicating a dysbiosis of the gut microbiome. Interestingly, the proportions of other genera such as *Lactobacillus* and *Desulfovibrio* were also affected by *P. gingivalis* administration [99]. Due to the changes in the gut microbiome, *P. gingivalis* administration leads to increased systemic inflammation, endotoxin levels, and intestinal permeability, even after a single exposure [100]. The increased systemic inflammation contributes to the changes in gene expression profiles of epididymal adipose tissue, increasing proinflammatory genes while decreasing insulin sensitivity [97], as well as changes in expression of genes associated with glucose and lipid metabolism [99,101]. However, the limitations associated with the use of mouse models should be mentioned. Mouse models lack some human-specific gut bacteria and proportion of bacterial phyla is different compared to humans. Furthermore, the immune responses also differ [102]. Altogether, these limitations indicate the need of human studies. In humans, the Human Microbiome Project demonstrated that several genera, including *Bacteroides, Faecalibacterium, Parabacteroides, Eubacteium, Alistipes, Dialister, Streptococcus, Prevotella, Rosburia, Coprococcus, Veillonella* and *Oscilibacter* could be detected in both the oral cavity and stool in more than 45% of the included patients [103], highlighting a potential role for oral bacteria in the disruption of the gut microbiome. It was therefore hypothesized that saliva is a key driver of microbial composition in the habitats above the stomach. However, the role of the continuous epithelial lining of the upper gastrointestinal mucosal surfaces has also been suggested [103].

Obesity is also associated with metabolic alterations such as glucose homeostasis including glucose intolerance, diabetes and insulin resistance [104], all of which are associated with low-grade inflammation. Metabolic endotoxemia induced by LPS has been associated with insulin resistance [105]. Interestingly, it was demonstrated that mice fed a high-fat diet and treated with antibiotics exhibited reduced inflammation, insulin resistance and fat mass gain, emphasizing a role of the gut microbiota [106]. In the context of periodontitis and experimental periodontitis induced by *P. gingivalis,* obese animals exhibited greater periodontal bone loss that in lean animals; a process attributed to a severe immune dysfunction [88,107,108,109]. Experimental periodontitis induced by multispecies (*P. gingivalis*, *F. nucleatum*, *P. intermedia*) oral gavage was associated with increased insulin resistance [110]. This modulation of the insulin resistance was linked to the recruitment of cells involved in the adaptive immune response, and a decrease in antibodies against *P. gingivalis* was associated with impaired glucose metabolism [110].

Conventional periodontal treatments including scaling and root planning with adjuvant therapies such as topical antibiotics have been demonstrated to alleviate some of the symptoms of metabolic disorders. In fact, such treatments have led to a reduction in serum glucose and glycated hemoglobin, insulin resistance, and fasting glucose levels [111,112,113]. There is little understanding of the impact of periodontal treatment on the composition of the gut microbiome. Due to the ability of periodontal treatment to change the serum metabolome, the effects on the composition of the microbiome should be investigated.

## 6. Clinical Implications

Periodontal treatment aims to achieve resolution of gingival inflammation and a decrease in the pathogenic bacterial load [114]. It includes nonsurgical periodontal therapy consisting of oral hygiene instructions, scaling and root planning of affected teeth. In cases of severe lesions, anti-infective treatment may be used as an adjunctive to mechanical treatment. Accordingly, antiseptics, antibiotics, and local anti-infective treatments such as photodynamic therapy have been proposed to reduce the need for additional surgical treatment [115,116,117]. Such therapeutic approaches are effective in most clinical situations. However, in some patients, refractory lesions and recurrence leading ultimately to tooth loss could be observed [118,119], emphasizing the need for pharmacological approaches such as antibiotics.

Recently, the modulation of the biofilm composition has also been proposed as a therapeutic approach through the administration of beneficial microbes [120,121]. In this aspect, *Lactobacillus* strains, especially *L.reuteri*, were the most evaluated in human clinical settings. They showed a significant impact with a reduction in clinical attachment loss (−0.42 mm, *p* = 0.002) and gingival inflammation, as well as a significant reduction in bleeding on probing (−14.66%, *p* = 0.03) [121]. The mechanisms associated with oral administration of probiotics at the periodontal level are not fully elucidated yet. However, probiotic treatment has demonstrated an inhibitory effect towards bacteria present within periodontal biofilms such as *Streptococcus mutans*, *Streptococcus gordonii*, *Tannerella forsythia* and *Actinomyces naeslundi*. Interestingly, it was also demonstrated that the antimicrobial activity of *L. reuteri* was not associated with its viability, as even heat-killed bacteria or culture supernatant were effective, indicating a likely role for substances produced by the beneficial bacteria such as reutericyclin [122]. Interestingly, in rodent models, the restoration of a healthy gut flora concomitant with administration of probiotics such as *L. plantarum* was associated to cardioprotective effects, improved cardiac function and decreased inflammatory markers [123].

The gut symbiont *Akkermansia muciniphila* (*A. muciniphila*) has been evaluated as a new potential therapeutic tool for periodontitis. *A. muciniphila* has attracted growing interest due to its host-beneficial properties. In several rodent models, supplementation with *A. muciniphila* reduces obesity, insulin resistance, glucose intolerance, steatosis and gut permeability [124,125,126]. Such properties suggest *A. muciniphila* as a next-generation probiotic [127]. Furthermore, there has been additional focus on one of its membrane proteins, Amuc_1100 [128], demonstrating its ability to recapitulate the effect induced by live bacteria. Amuc_1100 interacts with TLR- 2 and -4 resulting in the improvement of gut barrier function and anti-inflammatory IL-10 secretion in mouse models [125,129]. In humans, a recent proof-of-concept exploratory study shows that the supplementation with *A. muciniphila* was safe and well tolerated. Most importantly, the administration of this gut commensal administration in human obese volunteers, notably in pasteurized form, contributed to body weight reduction in comparison with placebo treated patients, but also reduced the level of markers associated with liver dysfunction and inflammation such as fasting plasma insulin, total cholesterol and γGT [130]. In the context of periodontitis, administration of *A.muciniphila* in a murine model of experimental periodontitis was associated with improved periodontal healing [120]. This effect was associated with a modulation of *P. gingivalis* virulence by *A. muciniphila* in cocultures. As observed for *L. reuteri*, some studies showed the role of a secreted bacterial byproduct, Amuc_1100 in the anti-inflammatory properties [125]. The impact of the oral administration of *A. muciniphila* on the gut microbiome was also observed and associated with changes in the ratio of Firmicutes/Bacteroidetes, highlighting a potential influence of gut ecology on systemic inflammation levels [120].

## 7. Conclusions

The detrimental effect of periodontitis on other major systemic diseases with high social and financial consequences has been demonstrated. Modulation of the microbiome has emerged as an exciting potential treatment that could pave the way for a new approach to managing periodontal diseases, as well as their systemic effects. New research is needed to establish the molecular mechanisms involved, and to elucidate effects related to both local antimicrobial properties and systemic anti-inflammatory effects associated with the transition from a dysbiotic to a healthy gut microbiome. Additionally, studies are needed to determine the most effective probiotic organisms, strains, dosage, and routes of administration necessary to achieve optimal outcomes in each respective disease. With further research into the effects of modulation of the oral microbiome on systemic disease, promising new treatments for these chronic inflammatory diseases will emerge.
